# 2293. Examining social vulnerability and its effect on COVID-19 transmission in households

**DOI:** 10.1093/ofid/ofad500.1915

**Published:** 2023-11-27

**Authors:** Sara H Goodman, Alexandra Mellis, Carlos G Grijalva, H Keipp Talbot, Melissa Stockwell, Son H McLaren, Ellen Sano, Suchitra Rao, Edwin J Asturias, Huong McLean, Edward Belongia, Prasanthi Govindaranjan, Clea Sarnquist, Yvonne A Maldonado, Katherine Ellingson, Karen Lutrick, Natalie M Bowman, Sarah E Smith-Jeffcoat, Melissa A Rolfes, Jessica E Biddle, Jessica T Lin

**Affiliations:** Stanford University School of Medicine, San Jose, California; Centers for Disease Control and Prevention, Atlanta, GA; Vanderbilt University Medical Center, Nashville, Tennessee; Vanderbilt University Medical Center, Nashville, Tennessee; Columbia University Irving Medical Center, New York City, New York; Columbia University Irving Medical Center, New York City, New York; Columbia University Irving Medical Center, New York City, New York; University of Colorado School of Medicine, Aurora, Colorado; University of Colorado School of Medicine, Aurora, Colorado; Marshfield Clinic Research Institute, Marshfield, WI; Marshfield Clinic Research Institute, Marshfield, WI; Stanford University School of Medicine, San Jose, California; School of Medicine, Stanford University, Palo Alto, California; Stanford University, Stanford, California; University of Arizona, Tucson, Arizona; University of Arizona College of Medicine, Tucson, Arizona; University of North Carolina, Chapel Hill, North Carolina; Centers for Disease Control and Prevention, Atlanta, GA; Centers for Disease Control and Prevention, Atlanta, GA; Centers for Disease Control and Prevention, Atlanta, GA; University of North Carolina at Chapel Hill, Chapel Hill, North Carolina

## Abstract

**Background:**

Social vulnerability impacts the transmission of SARS-CoV-2 (SCV2) among household contacts. Understanding these correlates can inform interventions to prevent infection among close contacts. We examined whether the social vulnerability index (SVI), a composite measure of socioeconomic status, household characteristics, racial and ethnic minority status, and housing type and transportation, is associated with the risk of SCV2 infection among household contacts.

Overall Social Vulnerability Index Diagram

**Figure:**
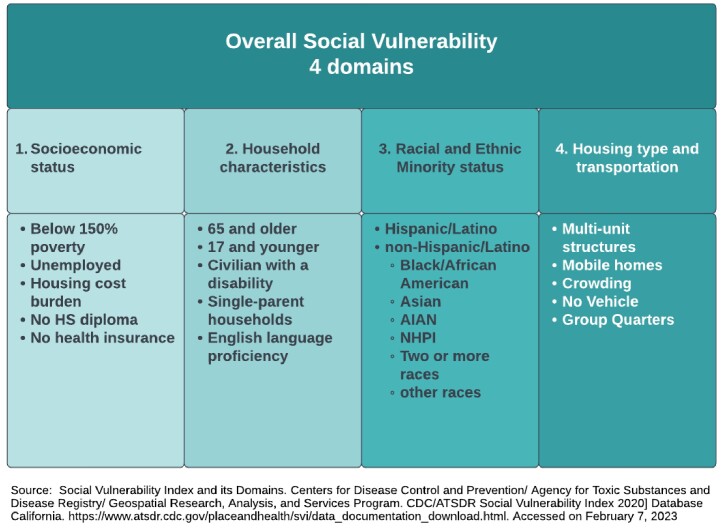

**Methods:**

We used data from a multi-site, prospective, case-ascertained household transmission study with daily nasal swabs for 10 days and RT-PCR testing to detect SCV2 infections in household contacts. Age and gender were self-reported and vaccination status was self-reported and verified. We mapped households to 2020 census tracts and the 2020 SVI (Figure 1). We examined the association between census tract-level SVI (in quartiles) and the risk of infection among household contacts using Poisson regression with generalized estimating equations, accounting for household clustering.

Inclusion criteria for analysis in this study.

**Figure:**
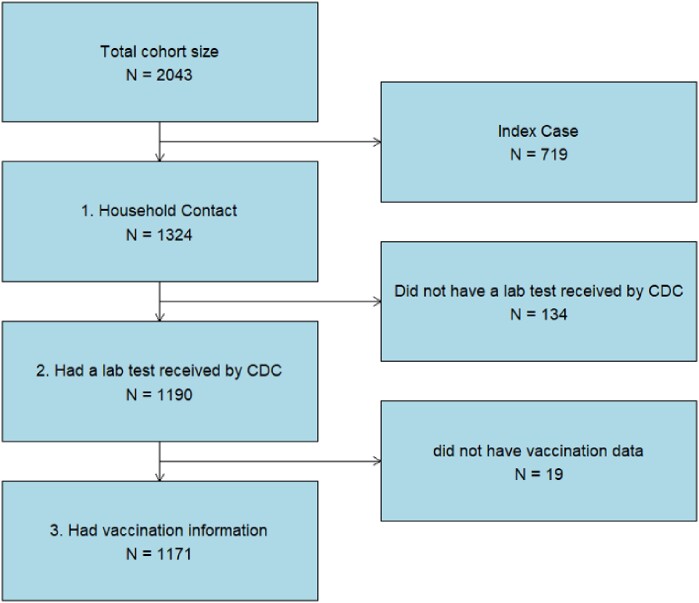

Inclusion criteria for analysis in this study.

**Results:**

Among 1,171 household contacts from 719 households, 67.4% developed SCV2 infection. After adjusting for the age of the contact and study site, contacts living in the most vulnerable SVI quartiles, Q3 (Incidence Rate Ratio [IRR] 1.19, 95% CI 1.01-1.40) and Q4 (IRR=1.18, 95% CI 1.00-1.40), had higher rates of infection compared to those living in the least vulnerable quartile (Q1) at the census tract level. To describe the effect of SVI accounting for vaccination, we performed a second regression adjusting for vaccine receipt among participants. We found that Q3 (IRR 1.19, 95% CI 1.01-1.40) still had higher rates of infection compared to those living in the least vulnerable quartile (Q1). Q4 was directionally consistent but confidence bounds crossed 1 (IRR=1.17, 95% CI 0.99-1.39).

**Figure:**
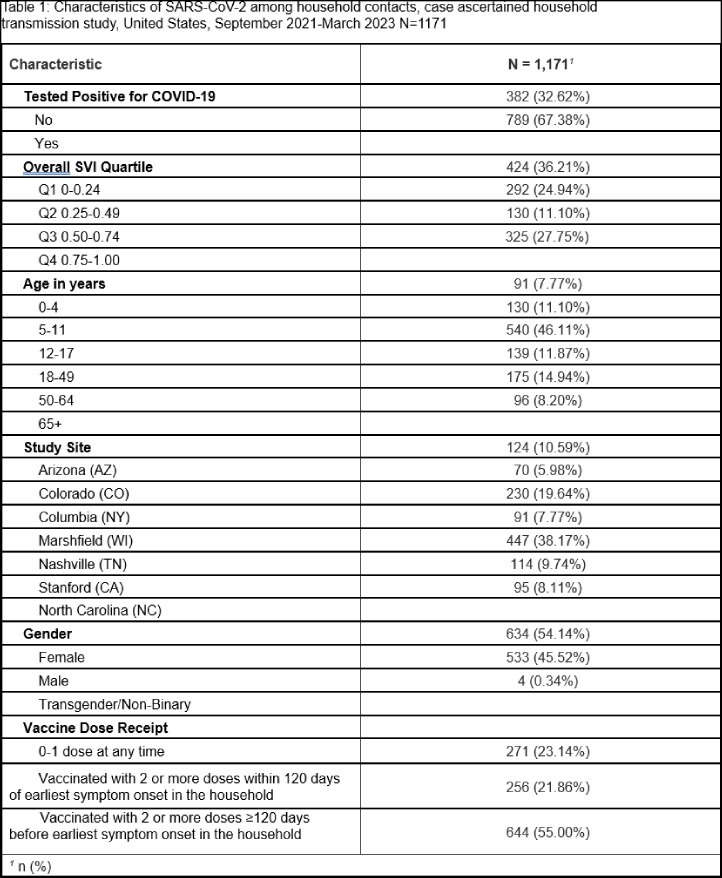

**Figure:**
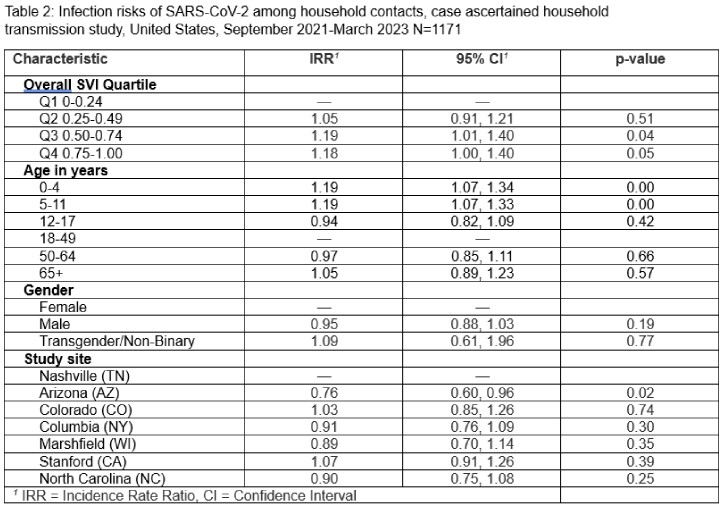

**Figure:**
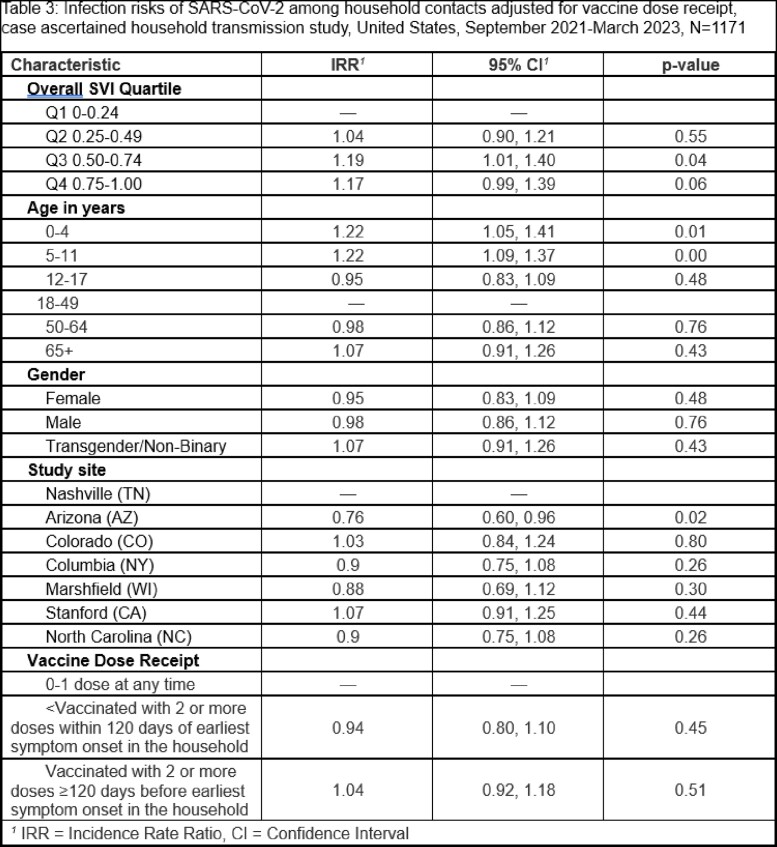

**Conclusion:**

Household contacts from census tracts with greater social vulnerability at the census tract level had a greater risk of SCV2 infection. These risks held even after accounting for vaccine receipt among participants. Future public health interventions should focus on reducing infection and transmission among individuals living in areas with higher social vulnerability beyond vaccination coverage.

**Disclosures:**

**Carlos G. Grijalva, MD, MPH**, AHRQ: Grant/Research Support|CDC: Grant/Research Support|FDA: Grant/Research Support|Merck: Advisor/Consultant|NIH: Grant/Research Support|Syneos Health: Grant/Research Support **Suchitra Rao, MBBS, MSCS**, Sequiris: Advisor/Consultant **Edwin J. Asturias, MD**, Hillevax: Advisor/Consultant|Moderna: Advisor/Consultant|Pfizer: Grant/Research Support **Huong McLean, PhD, MPH**, Seqirus: Grant/Research Support **Edward Belongia, MD**, Seqirus: Grant/Research Support **Yvonne A. Maldonado, MD**, Pfizer: Grant/Research Support|Pfizer: Site Investigator, DSMB member

